# Persistence at 24 months with denosumab among postmenopausal women with osteoporosis: results of a prospective cohort study

**DOI:** 10.1007/s11657-018-0491-z

**Published:** 2018-08-07

**Authors:** Stuart L. Silverman, E. Siris, D. Belazi, C. Recknor, A. Papaioannou, J. P. Brown, D. T. Gold, E. M. Lewiecki, G. Quinn, A. Balasubramanian, S. Yue, B. Stolshek, D. L. Kendler

**Affiliations:** 1 OMC Clinical Research Center, Cedars-Sinai Medical Center and David Geffen School of Medicine UCLA, 8641 Wilshire Blvd, Suite 301, Beverly Hills, CA 90211 USA; 2 Columbia University Medical Center, 180 Fort Washington Avenue, HP9-964, New York, NY USA; 3 AlchemiPharma, 1582 High Grove LN, Malvern, PA USA; 4 United Osteoporosis Centers, 2350 Limestone Parkway, Gainesville, GA USA; 5 Juravinski Research Center, McMaster University, Room 151, 88 Maplewood Avenue, Hamilton, Canada; 6 CHU de Québec (CHUL) Research Center, Laval University, Room TR-83, 2705 Laurier Boulevard, Quebec City, QC Canada; 7 Duke University Medical Center, Box 3003, Durham, NC USA; 8 New Mexico Clinical Research and Osteoporosis Center, University of New Mexico School of Medicine, 300 Oak St. NE, Albuquerque, NM USA; 9 Outlier Statistics Ltd, 25 Blacksmith Close, St Michaels Mead, Bishop’s Stortford, UK; 10 Amgen Inc., One Amgen Center Drive, Thousand Oaks, CA USA; 11 Department of Medicine, University of British Columbia, Prohealth, 150-943 W Broadway, Vancouver, BC Canada

**Keywords:** Persistence, Denosumab, Cohort study, Osteoporosis, Clinical practice, Bone mineral density

## Abstract

**Summary:**

Persistence with prescribed medications for chronic diseases is important; however, persistence with osteoporosis treatments is historically poor. In this prospective cohort study of postmenopausal women treated for osteoporosis in real-world clinical practice settings in the USA and Canada, 24-month persistence with denosumab was 58%.

**Purpose:**

Patients who persist with their prescribed osteoporosis treatment have increased bone mineral density (BMD) and reduced risk of fracture. Twelve-month persistence with denosumab in routine clinical practice is as high as 95%, but there are limited data on longer-term persistence with denosumab in this setting.

**Methods:**

This single-arm, prospective, cohort study evaluated 24-month persistence with denosumab administered every 6 months in postmenopausal women receiving treatment for osteoporosis in real-world clinical practice in the USA and Canada. Endpoints and analyses included the percentage of patients who persist with denosumab at 24 months (greater than or equal to four injections with a gap between injections of no more than 6 months plus 8 weeks), the total number of injections received by each patient, changes in BMD in persistent patients, and the incidence of serious adverse events (SAEs) and fractures.

**Results:**

Among 935 enrolled patients, 24-month persistence was 58% (50% in US patients and 75% in Canadian patients). A majority of patients received at least four injections over the observation period (62% of US patients and 81% of Canadian patients). Among patients who were persistent at 24 months and who had a baseline, 12-month, and 24-month DXA scan, mean BMD increased from baseline to 24 months by 7.8% at the lumbar spine and 2.1% at the femoral neck. SAEs and fractures were reported for 122 (13.0%) patients and 54 (5.8%) patients, respectively.

**Conclusions:**

Persistence with denosumab for 24 months yields improvement in BMD among postmenopausal women with osteoporosis treated in routine clinical practice in the USA and Canada.

**Electronic supplementary material:**

The online version of this article (10.1007/s11657-018-0491-z) contains supplementary material, which is available to authorized users.

## Introduction

Osteoporosis is a chronic disease that requires long-term pharmacological treatment. Patients who are persistent or continue with their prescribed osteoporosis treatment have increased bone mineral density (BMD) and reduced risks of fracture, hospitalization, and morbidity [1–3]. When osteoporosis treatments are used inconsistently, fracture risk can increase in nonpersistent (versus persistent) patients, especially for reversible drugs that are metabolized and eliminated [4, 5].

Historically, persistence with osteoporosis therapies has been challenging regardless of dosing interval or route of administration. A high proportion of patients who are prescribed oral bisphosphonates discontinue treatment within their first year of therapy, and there is considerable variation in persistence among studies, with the percentage of patients who persist at 12 months varying from 13 to 63% [6, 7]. The introduction of oral bisphosphonates requiring less frequent administration (weekly rather than daily dosing regimens) has led to improvements in persistence [8, 9]; however, studies often report 12-month persistence below 50%, regardless of dosing options [8, 10–12]. Inconsistent or low rates of persistence are not limited to oral osteoporosis therapies and have also been observed for intravenous zoledronic acid and for teriparatide [13–18].

Persistence data beyond 12 months are sparse; however, studies have consistently demonstrated that persistence continues to decline beyond the first year of therapy. For example, a claims database study and a prescription database study found that persistence with oral osteoporosis therapies declined from 41% after 2 years to only 3% after 12–14 years [19] and from 59% after 1 year to 25% after 5 years, respectively [20].

Denosumab (Prolia^®^; Amgen Inc., Thousand Oaks, CA, USA), administered as a subcutaneous injection once every 6 months, is a clinically effective therapy to reduce fracture risk in postmenopausal women with osteoporosis [21]. Twelve-month persistence with denosumab was shown to be high both in the clinical trial setting (93 and 94%) [22, 23] and in routine clinical practice (70–95%) [24–26]. There are limited data on persistence beyond 12 months with denosumab in the real-world setting, with nonintervention studies reporting 24-month persistence of 40–86% [24, 26–30].

Here, we report the results of a 24-month, prospective, cohort study of denosumab treatment among postmenopausal women with osteoporosis in the USA and Canada. The interim 12-month persistence data have been published previously [25]. The 24-month persistence data as well as the effect of persistence on BMD are presented.

## Methods

### Study design

Details of the study design have been published previously [25]. In brief, this was a prospective, multicenter, single-arm, cohort, 24-month study of persistence with denosumab administered once every 6 months in postmenopausal women with osteoporosis. Patients were enrolled at primary care and specialty practices in the USA and Canada.

This study was designed to reflect real-world treatment. Postmenopausal women who had received denosumab for the treatment of osteoporosis in accordance with the US or Canadian prescribing information were eligible for enrollment. Patients’ decision to receive denosumab was made before they signed the informed consent form, and patients were enrolled within 4 weeks following receipt of their first denosumab injection. Patients were excluded if they were currently participating in a denosumab clinical trial, if they had participated previously in a denosumab clinical trial, if they had participated in other drug or device clinical trials in the previous 6 months, or if treatment with denosumab was contraindicated in accordance with the US or Canadian prescribing information. Some patients may have been followed for more than 24 months due to timing of clinic visits.

### Data collection

Methods for data collection have been described previously for the interim 12-month analysis [25]. In brief, physicians at each site recorded patient information for routine clinical care, including medical history, demographics, BMD, fracture (osteoporotic or other), denosumab injections, previous and concomitant therapies, comorbidities, and serious adverse events (SAEs).

At enrollment, patients completed a baseline questionnaire about their health history, insurance, income, education, marital status, proximity to the clinical practice, and use of a denosumab support program. Compensation for completing the questionnaires was provided if permitted by regional laws or regulatory guidelines.

Dual-energy x-ray absorptiometry (DXA) assessments were not a requirement to participate in the study, but lumbar spine and femoral neck scans were collected when available. There was no central review and adjudication of DXA scans, which were performed in accordance with the standard procedures at each facility.

### Study endpoints

The study endpoints included persistence with denosumab, change from baseline in BMD in persistent patients, and the incidence of SAEs and fractures.

In this study, 12-month persistence and 24-month persistence were defined as patients who received at least two denosumab injections (12 months) or four denosumab injections (24 months), including one at study entry, with an interval between injections of no longer than 6 months plus 8 weeks. The total number of denosumab injections per patient was recorded.

Sensitivity analyses examined any effect on persistence with denosumab by varying the time interval between injections (6 months plus 4 weeks, 6 months plus 6 weeks, or 6 months plus 12 weeks). These time intervals were chosen based on the established duration of denosumab activity [31] and to account for potential challenges associated with return clinic visits, such as scheduling appointments and obtaining prior authorization for treatment.

### Statistical analyses

The planned overall sample size calculation has been described previously [25]. The analyses for this study are descriptive. Categorical outcomes are presented as number and percentage; continuous variables are presented as number, mean with standard deviation (SD), and median with interquartile range (Q1, Q3).

Patient demographics are summarized overall and by country of enrollment, and baseline characteristics are summarized by country of enrollment and by 24-month persistence with denosumab. Persistence at 12 and 24 months is reported as a percentage with 95% confidence intervals (CIs). Patients who discontinued denosumab treatment, did not have dosing information, or withdrew from the study were considered nonpersistent.

A post hoc exploratory analysis assessed BMD in patients who had baseline and postbaseline DXA scans taken within prespecified visit windows (baseline: up to 1 year prior or up to 3 months after baseline injection; postbaseline: DXA nearest to the dose date plus 366 days) at the same anatomical site (lumbar spine or femoral neck) using the same side (femoral neck) and machine type. Postbaseline DXA assessments were selected to match baseline for body side for femoral neck only. We restricted the BMD analysis to persistent patients, because the number of nonpersistent patients with baseline and follow-up DXA scans (six patients at 12 months and 18 patients at 24 months) was considered too small to provide meaningful data. The percentage change from baseline in BMD at the lumbar spine or femoral neck (presented as mean and 95% CIs), the percentage of patients who had ≥ 3% improvement from baseline in BMD at the lumbar spine or femoral neck, and the percentage of patients who had T-scores ≤ − 2.5 at the lumbar spine and femoral neck are reported for patients who were persistent at 24 months and who had a baseline, 12-month, and 24-month DXA scan.

SAEs were coded using the Medical Dictionary for Regulatory Activities (v.17.0) and tabulated by system organ class and preferred term.

Fracture data were collected as AEs and were not adjudicated. Fractures were defined as all fractures excluding fractures not associated with decreased BMD (skull, face, mandible, metacarpals, fingers, toes, and cervical vertebrae), pathologic fractures, and fractures associated with severe trauma (defined as a fall from a height higher than a stool, chair, or first rung of a ladder, or severe trauma other than a fall). All spine fractures were confirmed by radiological assessments.

## Results

Physicians across 80 practices (USA, 54; Canada, 26) recruited patients; physician and site characteristics have been described previously [25]. Patients were enrolled into the study between July 2011 and April 2012, and the last reported follow-up was in April 2014.

**Fig. 1:**
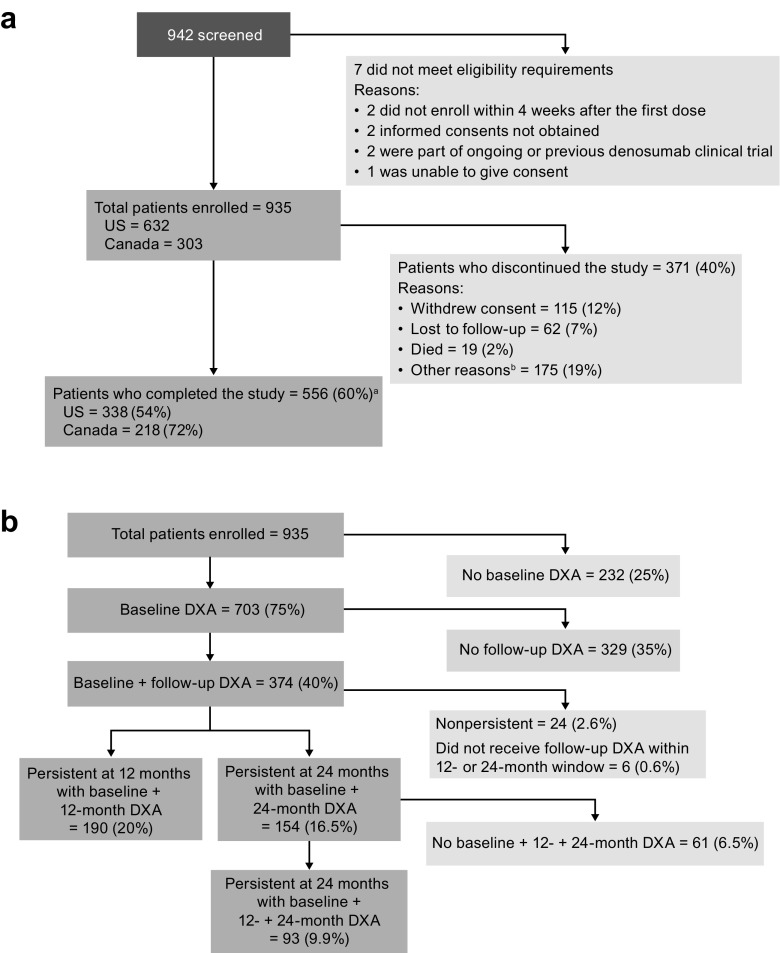
Flow of patients through the study. **a** Patients included in the analysis of persistence. Patient enrollment was completed on April 5, 2012, and the end of follow-up was on April 14, 2014. **a** Study completion data were missing for eight patients. **b** Provision of “other reasons” for discontinuation from the study was not required per protocol. **b** Patients included in the analysis of BMD. DXA scans were taken within prespecified visit windows (baseline: up to 1 year prior or up to 3 months after baseline injection; postbaseline: DXA nearest to the dose date plus 366 days)

The flow of patients through the study is presented in Fig. 1a. Of 942 patients screened, seven did not meet the eligibility criteria, and 935 were enrolled (USA, 632; Canada, 303). The primary reasons for study discontinuation were withdrawal of consent (115 [12.3%] patients) and “other” reasons (175 [18.7%] patients; not specified per protocol). Almost two-thirds of the overall patient population (556 patients, 59%) completed the study.

Among the enrolled patients, 703 (75%) had a baseline DXA scan, and 374 (40%) had a baseline and follow-up DXA scan (Fig. 1b). Twenty-four patients were nonpersistent, and six did not receive their follow-up scan within the 12- or 24-month time window; these patients were excluded from the BMD analysis. There were 190 patients (20%) who were persistent at 12 months with a baseline and 12-month DXA scan and 154 patients (16.5%) who were persistent at 24 months with a baseline and 24-month DXA scan. Ninety-three patients (9.9%) who were persistent at 24 months had a baseline, 12-month, and 24-month DXA scan.

Study participants were observed for a median (Q1, Q3) of 24.3 (21.2, 25.3) months—24.6 (18.7, 26.0) months for US patients and 24.0 (23.4, 24.6) months for Canadian patients. Overall, 80% of patients (USA, 76%; Canada, 87%) remained in the study for at least 18 months.

### Patient demographics and baseline characteristics

The baseline characteristics of the overall, US, and Canadian patient populations are shown in Table 1. The mean (SD) age of the overall study population was 70.8 (9.9) years; 71% were at least 65 years old, and most (95%) were White. Mean (SD) baseline BMD T-scores at the lumbar spine and femoral neck for the overall study population were − 2.0 (1.3) and − 2.2 (0.9), respectively; 16% of patients had prior vertebral fractures, and 42% had prior nonvertebral fractures. Most patients (93%) had used an osteoporosis therapy before enrollment, 59% for more than 5 years. The baseline characteristics of persistent and nonpersistent subjects within each country were similar (Supplementary Material).

**Table 1:** Patient demographics and baseline characteristics

Characteristics	USA (*N* = 632)	Canada (*N* = 303)	Overall (*N* = 935)
Age, years, mean (SD)	71.9 (10.0)	68.6 (9.2)	70.8 (9.9)
Age group, years, *n* (%)			
< 65	152 (24)	115 (38)	267 (29)
≥ 65 to < 75	216 (34)	102 (34)	318 (34)
≥ 75	264 (42)	86 (28)	350 (37)
Race, *n* (%)			
White	601 (95)	285 (94)	886 (95)
Asian	9 (1)	16 (5)	25 (3)
Black or African American	9 (1)	0 (0)	9 (1)
Other^a^	13 (2)	2 (0.7)	15 (2)
Body mass index, kg/m^2^			
Mean (SD)	25.5 (5.7)	26.1 (5.1)	25.7 (5.5)
≤ 25	351 (56)	127 (42)	478 (51)
> 25	269 (43)	155 (51)	424 (45)
Missing	12 (2)	21 (7)	33 (4)
Modified Wolfe comorbidity index, median (Q1, Q3)	2.0 (1.0, 3.0)	2.0 (1.0, 3.0)	2.0 (1.0, 3.0)
Number of prescription medications taken at baseline, median (Q1, Q3)	8.0 (5.0, 11.0)	5.0 (3.0, 7.0)	7.0 (4.0, 10.0)
Lumbar spine T-score			
Mean (SD)	− 1.9 (1.4)	− 2.1 (1.3)	− 2.0 (1.3)
≤ − 2.5, *n* (%)	221 (40)	115 (46)	336 (42)
> − 2.5, *n* (%)	328 (60)	134 (54)	462 (58)
Femoral neck T-score			
Mean (SD)	− 2.3 (0.8)	− 1.9 (1.0)	− 2.2 (0.9)
≤ − 2.5, *n* (%)	262 (47)	69 (28)	331 (41)
> − 2.5, *n* (%)	300 (53)	176 (72)	476 (59)
History of fracture^b^, *n* (%)	325 (51)	145 (48)	470 (50)
Vertebral	104 (16)	45 (15)	149 (16)
Nonvertebral^c^	271 (43)	120 (40)	391 (42)
Time since the most recent osteoporotic fracture, *n* (%)			
< 12 months	40 (6)	23 (8)	63 (7)
≥ 12 months	283 (45)	122 (40)	405 (43)
Parental history of hip fracture, *n* (%)	127 (20)	69 (23)	196 (21)
Osteoporosis medication			
Any exposure to osteoporosis therapy prior to enrollment, *n* (%)	587 (93)	280 (92)	867 (93)
Use of osteoporosis therapy > 5 years prior to enrollment, *n* (%)	360 (57)	187 (62)	547 (59)
Number of prior osteoporosis medications taken, mean (SD)	2 (1.3)	2 (1.2)	2 (1.3)

Q1, Q3 means interquartile range

*SD* standard deviation

^a^Other includes mixed race, American Indian or Alaska Native, Native Hawaiian or other Pacific Islander, or others (not specified)

^b^Excludes fractures not associated with decreased bone mineral density (skull, face, mandible, metacarpals, fingers, toes, and cervical vertebrae), pathologic fractures, and fractures associated with severe trauma (defined as a fall from a height higher than a stool, chair, or first rung of a ladder, or severe trauma other than a fall)

^c^Includes fractures of the pelvis, hip, lower leg (not knee or ankle), ribs, shoulder, forearm, and wrist and excludes pathologic fractures and fractures associated with severe trauma

### 24-month persistence with denosumab

**Fig. 2:**
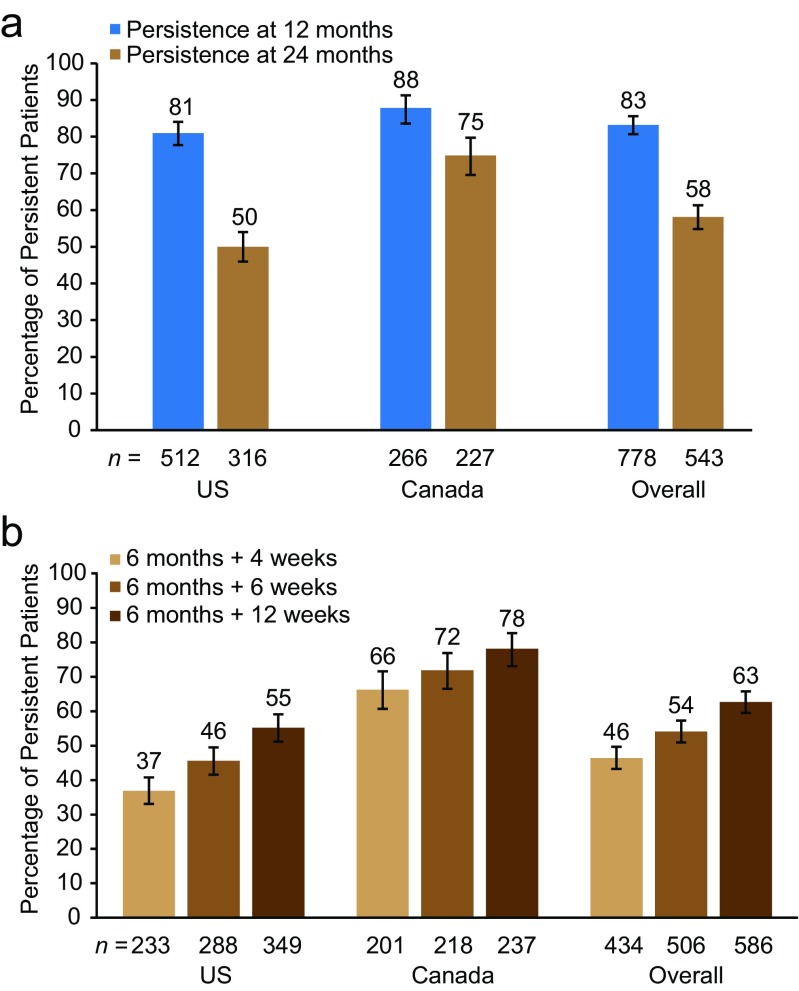
Persistence with denosumab at 12 and 24 months. **a** Percentage of patients who were persistent with denosumab at 12 and 24 months. The primary analysis set included patients who received at least two denosumab injections (12-month persistence) or four denosumab injections (24-month persistence), with an injection interval of no longer than 6 months plus 8 weeks. **b** Sensitivity analysis of 24-month persistence. Sensitivity analyses were done to evaluate whether persistence is affected by varying the time interval between injections—6 months plus 4 weeks, 6 months plus 6 weeks, or 6 months plus 12 weeks. Error bars represent 95% confidence intervals

The percentage of patients (95% CI) who persisted with denosumab at 24 months (greater than or equal to four injections with a dosing interval within 6 months plus 8 weeks) was 58% (55–61%) for the overall study population, 50% (46–54%) for the US population, and 75% (70–80%) for the Canadian population (Fig. 2a). In sensitivity analysis, the persistence with denosumab at 24 months increased with longer intervals between injections, overall and within each country (Fig. 2b).

### Number of denosumab injections

The median (Q1, Q3) number of denosumab injections received was 5 (3, 5) overall, 4 (3, 5) in the USA, and 5 (4, 5) in Canada; a majority of patients received at least four injections over the observation period (overall, 68%; USA, 62%; Canada, 81%; Table 2).

**Table 2:** Number of injections received by patients

	USA (*N* = 632)	Canada (*N* = 303)	Overall (*N* = 935)
Number of injections per subject, median (Q1, Q3)	4 (3, 5)	5 (4, 5)	5 (3, 5)
Patients who received injections over the observation period, *n* (%)			
1 injection	80 (13)	24 (8)	104 (11)
2 injections	76 (12)	16 (5)	92 (10)
3 injections	82 (13)	19 (6)	101 (11)
4 injections	121 (19)	36 (12)	157 (17)
5 injections	273 (43)	208 (69)	481 (51)

Q1, Q3 means interquartile range

### BMD

**Fig. 3:**
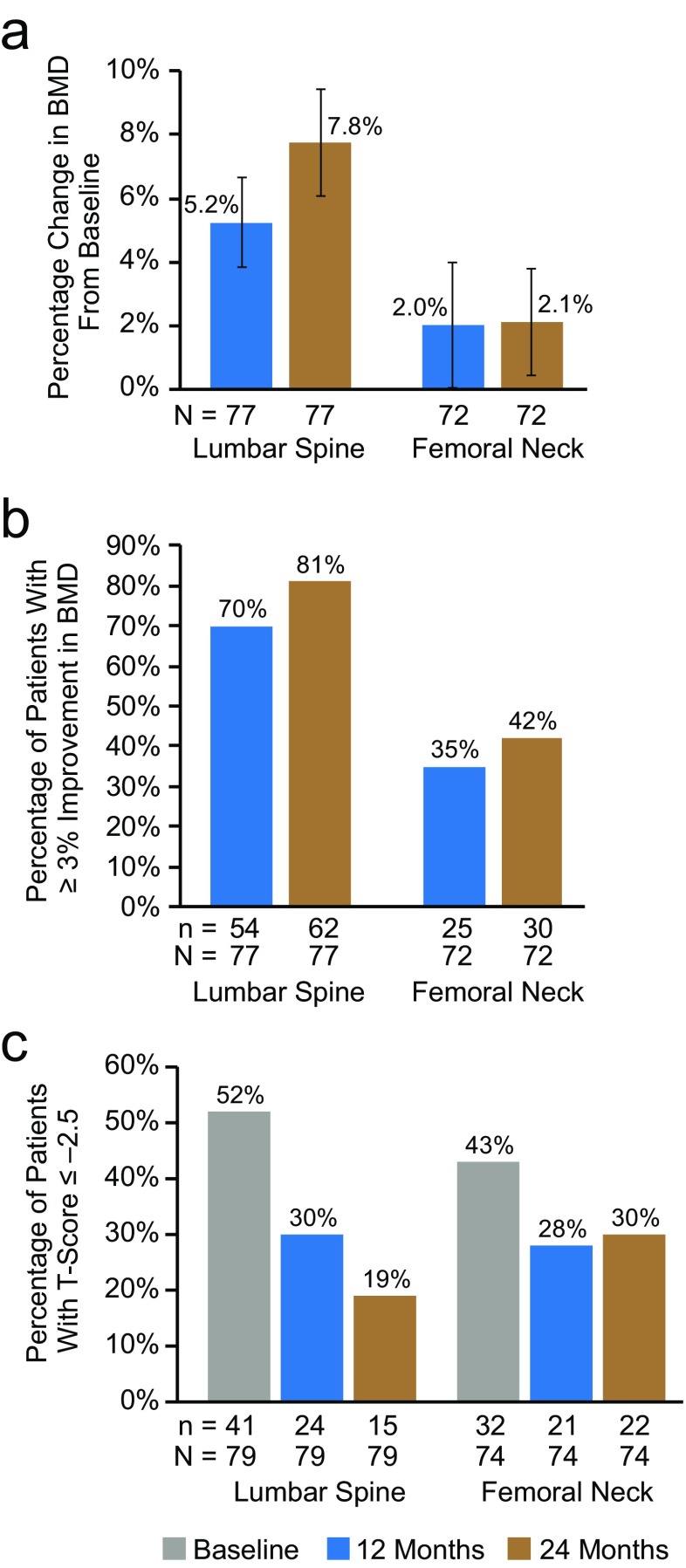
BMD at the lumbar spine and femoral neck in patients who were persistent at 24 months and who had a baseline, 12-month, and 24-month DXA scan. **a** Mean percentage change in BMD from baseline. Error bars represent 95% confidence intervals; 77 and 72 patients were evaluable at the lumbar spine and femoral neck, respectively. **b** Proportion of patients with ≥ 3% improvement in BMD at 12 and 24 months; 77 and 72 patients were evaluable at the lumbar spine and femoral neck, respectively. **c** Proportion of patients with T-scores ≤ − 2.5 at baseline, 12 months, and 24 months; 79 and 74 patients were evaluable at the lumbar spine and femoral neck, respectively. *BMD* bone mineral density; *DXA* dual-energy x-ray absorptiometry

We conducted the analysis of BMD on patients who were persistent at 24 months and who had a baseline, 12-month, and 24-month DXA scan. The mean (95% CI) percentage change in BMD from baseline at months 12 and 24 was 5.2% (3.8–6.6%) and 7.8% (6.1–9.4%) at the lumbar spine and 2.0% (0.01–4.0%) and 2.1% (0.4–3.8%) at the femoral neck, respectively (Fig. 3a). In addition, the percentage of patients who had a ≥ 3% improvement in BMD at months 12 and 24 was 70 and 81% at the lumbar spine and 35 and 42% at the femoral neck, respectively (Fig. 3b). Fifty-two percent of this subpopulation of patients had osteoporotic T-scores (≤ − 2.5) at the lumbar spine at baseline, decreasing to 19% at 24 months (Fig. 3c). Likewise, the percentage of patients with osteoporotic T-scores at the femoral neck decreased from 43% at baseline to 30% at 24 months (Fig. 3c).

The number of nonpersistent patients with a baseline and evaluable DXA BMD at 12 and 24 months was only 6 and 18, respectively, and therefore, too small to make any meaningful comparisons.

### Safety

The patient incidence of SAEs was 13.0% (122/935), with 15.0% (95/632) for the USA and 8.9% (27/303) for Canada (Table 3). SAEs led to discontinuation of denosumab in eight (0.9%) patients and withdrawal from the study in four (0.4%) patients. The most frequently reported SAEs (≥ 0.5%) were pneumonia (10 [1.1%] patients), cellulitis (five [0.5%] patients), acute myocardial infarction (five [0.5%] patients), and breast cancer (five [0.5%] patients). No SAEs of osteonecrosis of the jaw, atypical femoral fracture, fracture healing complications, hypocalcemia, eczema, or those potentially associated with hypersensitivity were reported. Fatal AEs were reported in 2.0% of patients overall, but none was considered treatment-related by the treating physicians.

**Table 3:** SAE and fractures

Event	USA (*N* = 632), *n* (%)	Canada (*N* = 303), *n* (%)	Overall (*N* = 935), *n* (%)
SAEs	95 (15.0)	27 (8.9)	122 (13.0)
Leading to discontinuation of denosumab	5 (0.8)	3 (1.0)	8 (0.9)
Leading to withdrawal from the study	4 (0.6)	0 (0)	4 (0.4)
Fractures^a^	43 (6.8)	11 (3.6)	54 (5.8)
Fatal AEs^b^	15 (2.4)	4 (1.3)	19 (2.0)

*AE* adverse event, *SAE* serious AE

^a^Excludes fractures not associated with decreased bone mineral density (skull, face, mandible, metacarpals, fingers, toes, and cervical vertebrae), pathologic fractures, and fractures associated with severe trauma (defined as a fall from a height higher than a stool, chair, or first rung of a ladder, or severe trauma other than a fall)

^b^Fatal AEs for the overall patient population were infections (five patients), cardiac disorders (four patients), deaths (three patients), cancer (three patients), respiratory disorders (two patients), lupus (one patient), and arteriosclerosis (one patient)

Fractures were reported for 5.8% (54/935) of patients, with 6.8% (43/632) in the USA and 3.6% (11/303) in Canada. The most frequently reported sites of fracture were the foot (15 [1.6%] patients—10 metatarsus and four tarsus) and the spine (11 [1.2%] patients—seven lumbar vertebrae and four thoracic vertebrae). Among patients who had fractures, all 11 in Canada and 21 of 43 patients in the USA were persistent at 24 months. The low number of fractures precluded an analysis of fracture incidence in persistent versus nonpersistent patients.

## Discussion

Persistence with prescribed medications is essential to achieve and maintain positive clinical outcomes. This prospective cohort study of postmenopausal women in the USA and Canada who received denosumab for the treatment of osteoporosis as part of routine clinical care provides insights into persistence with denosumab at 24 months as well as increases in BMD in persistent patients.

In our study, 58% of patients were persistent with denosumab after 24 months of treatment, compared with 82% at 12 months [25], and as expected, 24-month persistence increased as the permissible time interval between injections was increased. The percentage of patients (58%) in the overall patient population who persisted with denosumab at 24 months is higher than that observed in a previous retrospective observational study (41%) [26]. This percentage is also higher than 24-month persistence observed with other postmenopausal osteoporosis medications in the prospective, observational POSSIBLE US study (46.2%) [32].

It is not unexpected that the persistence of osteoporosis medications in real-world settings is lower than that observed in clinical trials, because in the clinical trial setting, patients interact regularly with clinical trial staff at fixed time intervals. Some of the reasons cited for poor persistence with osteoporosis medications in clinical practice include inconvenience of treatment, out-of-pocket costs, AEs, lack of appropriate patient education/knowledge, and the interaction and support provided by the physicians and healthcare team [33–35].

Although our study was not designed to compare 24-month persistence between two countries, the 25-percentage point difference between the USA and Canada (USA, 50%; Canada, 75%) is noteworthy. Previous retrospective claims-based analyses have shown that 24-month persistence with denosumab can vary by country, with observed incidences of 63% in Canada [27], 41% in the USA [26], and between 40 and 86% in Europe [24, 28, 29]. Patient support programs were available in both countries, but site participation was voluntary. Support programs can include educational materials and reminder calls. Such programs may be sponsored by the manufacturer or part of normal office practice. Patient participation in a support program was documented at enrollment, but limited data were collected on the use of such programs during the study.

Differences in healthcare systems between the two countries may have contributed to the observed results. Canada has a publicly funded healthcare system, and at the time of this study, patients may have received denosumab at no cost, paid out of pocket, or been reimbursed by extended medical insurance coverage. In the USA, denosumab may have been covered either by the Centers for Medicare and Medicaid Services or by an individual’s medical insurance, and a patient may have incurred considerable out-of-pocket expenses for treatment. Having to pay for treatment at the time of this study may have resulted in lower long-term persistence in the US population, recognizing that denosumab availability and reimbursement policies may change over time.

Long-term use of denosumab in the clinical trial setting has demonstrated continuous improvement in BMD as well as reductions in fracture risk [36]. In addition, a meta-analysis of 12 studies showed that nonpersistence with osteoporosis medications increased fracture risk by 30–40% relative to persistent patients [5], and a US claims-based analysis of oral bisphosphonates demonstrated that persistent patients were 26% less likely to have a fracture diagnosis claim than those who were nonpersistent [4]. Among the subset of patients in our study who were persistent at 24 months and had baseline, 12-month, and 24-month DXA scans, a majority of patients had ≥ 3% improvement in BMD from baseline to month 24 at the lumbar spine, and almost one half of patients had ≥ 3% improvement in BMD from baseline to month 24 at the femoral neck. Although the number of nonpersistent patients with follow-up DXA scans was too low to provide a meaningful comparison, these data underscore the importance of persistence with denosumab treatment to achieve optimal therapeutic benefits in the clinical practice setting.

According to a Call to Action Summary from the American Society for Bone and Mineral Research in 2016 [37], patients are increasingly reluctant to take osteoporosis therapies, reporting fears of uncommon side effects, such as atypical femur fractures and osteonecrosis of the jaw [38]. The overall incidence of AEs in our study was consistent with previous denosumab studies [21–23, 31, 39, 40], and no new safety signals were identified. Other factors contributing to nonpersistence with osteoporosis medications include inconvenient or complex dosing regimens, lack of understanding of the benefits of therapy, and cost [3, 35, 41–43]. Osteoporosis is not the only chronic disease for which there is suboptimal persistence with therapy. For example, persistence with new drug therapy across six chronic conditions (glaucoma, lipid metabolism disorders, osteoporosis, diabetes mellitus, overactive bladder, and hypertension) declined between 10 and 15 percentage points over the course of 6 months and continued to decline over 2 years of follow-up [44].

The main strength of our study is that it evaluated persistence with denosumab over 24 months in a large cohort of patients from a wide range of clinical practice types in real-world community settings in North America. There are, however, a number of limitations. A high proportion of patients had a history of fracture and were of older age, reflecting a population at high risk of fracture who could benefit from denosumab treatment; however, the majority of patients were White, limiting the generalizability of the results. We did not collect data on how denosumab was provided to the patients (prescription versus physician-administered and reimbursed). In addition, patient enrollment began only 1 year after denosumab was approved in both countries; therefore, physicians who participated in this study may have been early adopters and more willing to prescribe a new medication to their patients. Furthermore, patients agreed to participate in the study; they were aware that their medication-taking behavior was being observed, and approximately one-half participated in voluntary denosumab patient support programs, all of which may have influenced persistence. It should be noted that we did not collect data on the consequences of discontinuing denosumab―denosumab is a reversible agent, and cessation of long-term denosumab therapy is associated with decreases in BMD and transient increases in bone turnover markers [45], and multiple vertebral fractures have been observed after treatment cessation in some patients, with risk appearing to be higher in patients that have a history of vertebral fracture [30].

In summary, more than one-half of women with osteoporosis treated with denosumab in a real-world clinical practice setting were persistent with denosumab at 24 months. In addition, patients who persisted with denosumab beyond 12 months had further improvements in BMD. Further studies are needed to explore the factors that influence persistence with denosumab, with the long-term goal of further improving patient outcomes.

## Electronic supplementary material

ESM 1 (DOCX 23.7 kb)
