# Supplementary material for: Persistence at 24 months with denosumab among postmenopausal women with osteoporosis: results of a prospective cohort study
Source: Arch Osteoporos. 2018 Aug 7;13(1):85. doi: 10.1007/s11657-018-0491-z (PMC6096691; doi:10.1007/s11657-018-0491-z)
Supplement: Supplementary file 1 — (DOCX 23.7 kb) [file 11657_2018_491_MOESM1_ESM.docx]

## Electronic Supplementary Material

## Persistence at 24 months with denosumab among postmenopausal women with osteoporosis: results of a prospective cohort study

***Osteoporosis International***

S.L. Silverman, E. Siris, D. Belazi, C. Recknor, A. Papaioannou, J.P. Brown, D.T. Gold, E.M. Lewiecki, G. Quinn, A. Balasubramanian, S. Yue, B. Stolshek, D.L. Kendler

Corresponding Author:

Stuart L. Silverman, MD, FACP, FACR

OMC Clinical Research Center, Cedars-Sinai Medical Center and David Geffen School of Medicine UCLA, 8641 Wilshire Blvd, Suite 301, Beverly Hills, CA 90211, USA

E-mail: [stuarts@BHillsRA.com](mailto:stuarts@BHillsRA.com)

## Online Resource Baseline characteristics by country and by 24-month persistence with denosumab

|  | **USA** | | **Canada** | |
| --- | --- | --- | --- | --- |
|  | **Persistent at 24 Months (*N* = 316)** | **Nonpersistent at 24 Months (*N* = 316)** | **Persistent at 24 Months (*N* = 227)** | **Nonpersistent at 24 Months (*N* = 76)** |
| Age group, years, *n* (%) | | | | |
| <65 | 81 (25.6) | 71 (22.5) | 87 (38.3) | 28 (36.8) |
| ≥65 to <75 | 122 (38.6) | 94 (29.7) | 77 (33.9) | 25 (32.9) |
| ≥75 | 113 (35.8) | 151 (47.8) | 63 (27.8) | 23 (30.3) |
| Race, *n* (%) | | | | |
| White | 300 (94.9) | 301 (95.3) | 214 (94.3) | 71 (93.4) |
| All others^a^ | 16 (5.1) | 15 (4.7) | 13 (5.7) | 5 (6.6) |
| Body mass index, kg/m^2^, *n* (%) | | | | |
| ≤25 | 175 (55.4) | 176 (55.7) | 90 (39.6) | 37 (48.7) |
| >25 | 135 (42.7) | 134 (42.4) | 125 (55.1) | 30 (39.5) |
| Missing | 6 (1.9) | 6 (1.9) | 12 (5.3) | 9 (11.8) |
| Number of prescription medications taken at baseline | | | | |
| Median (Q1, Q3) | 7.0 (5.0, 11.0) | 8.0 (6.0, 12.0) | 5.0 (3.0, 7.0) | 6.0 (3.0, 9.0) |
| Femoral neck T-score, *n* (%) | | | | |
| ≤–2.5 | 132 (41.8) | 130 (41.1) | 50 (22.0) | 19 (25.0) |
| >–2.5 | 158 (50.0) | 142 (44.9) | 134 (59.0) | 42 (55.3) |
| Missing | 26 (8.2) | 44 (13.9) | 43 (18.9) | 15 (19.7) |
| Lumbar spine T-score, *n* (%) | | | | |
| ≤–2.5 | 125 (39.6) | 96 (30.4) | 89 (39.2) | 26 (34.2) |
| >–2.5 | 163 (51.6) | 165 (52.2) | 100 (44.1) | 34 (44.7) |
| Missing | 28 (8.9) | 55 (17.4) | 38 (16.7) | 16 (21.1) |
| History of fracture,^b^ *n* (%) | | | | |
| Vertebral | 45 (14.2) | 59 (18.7) | 34 (15.0) | 11 (14.5) |
| Nonvertebral | 140 (44.3) | 131 (41.5) | 90 (39.6) | 30 (39.5) |
| Time since most recent previous fracture,^c^ months, *n* (%) | | | | |
| <12 | 14 (4.4) | 30 (9.5) | 22 (9.7) | 8 (10.5) |
| ≥12 | 167 (52.8) | 148 (46.8) | 101 (44.5) | 34 (44.7) |
| Missing | 135 (42.7) | 138 (43.7) | 104 (45.8) | 34 (44.7) |
| Parent fractured hip, *n* (%) | | | | |
| Yes | 63 (19.9) | 64 (20.3) | 53 (23.3) | 16 (21.1) |
| No | 222 (70.3) | 222 (70.3) | 144 (63.4) | 48 (63.2) |
| Unknown | 31 (9.8) | 30 (9.5) | 30 (13.2) | 12 (15.8) |
| Osteoporosis medication | | | | |
| Any exposure to prior prescription osteoporosis therapy prior to enrollment, *n* (%) | 296 (93.7) | 291 (92.1) | 209 (92.1) | 71 (93.4) |
| Use of osteoporosis therapy >5 years prior to enrollment, *n* (%) | 206 (65.2) | 154 (48.7) | 139 (61.2) | 48 (63.2) |
| Number of prior postmenopausal osteoporosis medications, mean (SD) | 2.1 (1.3) | 1.9 (1.3) | 1.9 (1.2) | 2.1 (1.3) |

*Q1, Q3* interquartile range, *SD* standard deviation.
*N* = number of subjects in the full analysis set.

Persistent at 24 months is defined as a subject who received four denosumab injections, with an interval between injections of no longer than 6 months plus 8 weeks.

^a^Includes American Indian or Alaska Native, Asian, Black or African American, mixed race, Native Hawaiian or Other Pacific Islander, and other.

^b^Excludes fractures not associated with decreased bone mineral density (skull, face, mandible, metacarpals, fingers, toes, and cervical vertebrae), pathologic fractures, and fractures associated with severe trauma (defined as a fall from a height higher than a stool, chair, or first rung of a ladder, or severe trauma other than a fall).

^c^Includes only subjects with previous fracture and non-missing fracture date.
